# Disease Biomarkers in Cerebrospinal Fluid of Patients with First-Onset Psychosis

**DOI:** 10.1371/journal.pmed.0030428

**Published:** 2006-11-07

**Authors:** Jeffrey T.-J Huang, F. Markus Leweke, David Oxley, Lan Wang, Nathan Harris, Dagmar Koethe, Christoph W Gerth, Brit M Nolden, Sonja Gross, Daniela Schreiber, Benjamin Reed, Sabine Bahn

**Affiliations:** 1 Institute of Biotechnology, University of Cambridge, Cambridge, United Kingdom; 2 Department of Psychiatry and Psychotherapy, University of Cologne, Cologne, Germany; 3 Proteomics Research Group, Babraham Institute, Cambridge, United Kingdom; 4 Ciphergen Biosystems Ltd., Surrey Research Park, Guildford, Surrey, United Kingdom; University of Queensland, Australia

## Abstract

**Background:**

Psychosis is a severe mental condition that is characterized by a loss of contact with reality and is typically associated with hallucinations and delusional beliefs. There are numerous psychiatric conditions that present with psychotic symptoms, most importantly schizophrenia, bipolar affective disorder, and some forms of severe depression referred to as psychotic depression. The pathological mechanisms resulting in psychotic symptoms are not understood, nor is it understood whether the various psychotic illnesses are the result of similar biochemical disturbances. The identification of biological markers (so-called biomarkers) of psychosis is a fundamental step towards a better understanding of the pathogenesis of psychosis and holds the potential for more objective testing methods.

**Methods and Findings:**

Surface-enhanced laser desorption ionization mass spectrometry was employed to profile proteins and peptides in a total of 179 cerebrospinal fluid samples (58 schizophrenia patients, 16 patients with depression, five patients with obsessive-compulsive disorder, ten patients with Alzheimer disease, and 90 controls). Our results show a highly significant differential distribution of samples from healthy volunteers away from drug-naïve patients with first-onset paranoid schizophrenia. The key alterations were the up-regulation of a 40-amino acid VGF-derived peptide, the down-regulation of transthyretin at ~4 kDa, and a peptide cluster at ~6,800–7,300 Da (which is likely to be influenced by the doubly charged ions of the transthyretin protein cluster). These schizophrenia-specific protein/peptide changes were replicated in an independent sample set. Both experiments achieved a specificity of 95% and a sensitivity of 80% or 88% in the initial study and in a subsequent validation study, respectively.

**Conclusions:**

Our results suggest that the application of modern proteomics techniques, particularly mass spectrometric approaches, holds the potential to advance the understanding of the biochemical basis of psychiatric disorders and may in turn allow for the development of diagnostics and improved therapeutics. Further studies are required to validate the clinical effectiveness and disease specificity of the identified biomarkers.

## Abstract 2

Protein profiles from 179 cerebrospinal fluid samples yield differences between patients with psychotic disorders and healthy volunteers, suggesting that such biomarkers could assist in the early diagnosis of mental illness.

## Editors' Summary

**Background.:**

Psychosis is an abnormal mental state characterized by loss of contact with reality, often associated with hallucinations, delusions, personality changes, and disorganized thinking. Psychotic symptoms occur in several psychiatric disorders, including schizophrenia, bipolar disorder, and psychotic depression. It is not clear what the underlying biological abnormalities in the brain are, and whether they are the same for the different psychotic illnesses. The hope is that recent advances in brain imaging and systematic characterization of genetic activity and protein composition in the brain might help to shed light on mental diseases, eventually leading to better diagnosis, treatment, and possibly even prevention.

**Why Was This Study Done?:**

This study was carried out in order to search for biomarkers for psychosis and schizophrenia by comparing the protein composition in the cerebrospinal fluid (the clear body fluid that surrounds the brain and the spinal cord) of patients with different psychotic disorders and normal individuals who served as controls.

**What Did the Researchers Do and Find?:**

The researchers used a technique called surface-enhanced laser desorption ionization mass spectrometry, which allows a comprehensive analysis of the protein composition of a particular sample, on a total of 179 cerebrospinal fluid samples. The samples came from 90 individuals without mental illness who served as controls, 58 people with schizophrenia who were very recently diagnosed and had not yet taken any medication, 16 patients with depression, five patients with obsessive-compulsive disorder, and ten patients with Alzheimer disease. All of the patients gave their informed consent to participate in the study. The researchers found that samples from treatment-naïve schizophrenic patients had a number of characteristic changes compared with samples from control individuals, and that those changes were not found in the patients with other mental illnesses. The researchers then wanted to test whether they would see the same pattern in a separate set of patients with schizophrenia versus controls, which turned out to be the case. Two of the changes in the cerebrospinal fluid that were associated with schizophrenia, namely higher levels of parts of a protein called VGF and lower levels of a protein called transthyretin, were also found in post-mortem brain samples of patients with schizophrenia compared with samples from controls. Lower levels of transthyretin were also found in serum (blood) of first-onset drug naïve schizophrenia patients.

**What Do These Findings Mean?:**

These results suggest that this approach has the potential to find biomarkers for psychosis and, possibly, schizophrenia that might help in the understanding of the molecular basis for these conditions. If shown, in future studies, to be directly involved in causing the disease symptoms, they would be important targets for treatment and prevention efforts, and might also be useful for diagnostic purposes. Overall, there are promising examples, such as this study, suggesting that new molecular techniques can yield fresh insights into psychiatric illnesses such as schizophrenia and other psychotic disorders. Additional studies are needed to confirm the findings presented here and to address many open questions, and would seem well justified given these results.

**Additional Information.:**

Please access these Web sites via the online version of this summary at http://dx.doi.org/10.1371/journal.pmed.0030428.

- MedlinePlus entries on psychosis and schizophrenia
- The National Alliance for Research on Schizophrenia and Depression
- The National Alliance for the Mentally Ill
- The Schizophrenia Society of Canada
- Wikipedia entries on psychosis and schizophrenia (note that Wikipedia is an online encyclopedia that anyone can edit)

## Introduction

Schizophrenia is the most devastating and enduring psychotic disorder affecting as many as 1% of the population worldwide, with a similar prevalence between the sexes and throughout diverse cultures and geographic areas [1,2]. To date, the etiology of schizophrenia remains elusive. The disorder is almost certainly the result of a complex interaction between numerous predisposing genes and environmental factors. Many studies have focused on the identification of schizophrenia “risk genes”/genetic polymorphisms, and although such “risk genes” may play a role in a small number of schizophrenia cases, the great majority of patients do not exhibit any apparent polymorphisms [3]. An alternative approach to genetic studies is to screen for disease markers (biomarkers). Biomarkers or biomarker signatures are indicators of a disease state that are usually linked to an ongoing pathophysiology and thus may also provide information and insights into the underlying molecular mechanisms of a given disease. Typical examples include diabetes and cardiovascular disorders, where increased glucose and low-density lipoprotein levels, respectively, are hallmark biomarkers for the diseases. Both cardiovascular disorders and diabetes (particularly Type II diabetes) are very similar to schizophrenia in that they are a result of genetic and environmental factors. Thus, even if the etiologies for schizophrenia and other psychotic disorders remain unknown, a biomarker or biomarker signature that accurately identifies the clinical syndrome would allow for improved diagnosis, prognosis, and disease monitoring as well as the development of novel therapeutic approaches.

Surface-enhanced laser desorption ionization (SELDI) mass spectrometry is a powerful tool for identifying a characteristic “fingerprint” of proteins and peptides in body fluids and tissues for a given condition, e.g., drug treatments and diseases (for review, see [4]). This technology utilizes modified surfaces to capture proteins/peptides, while a time-of-flight mass spectrometer is used to quantitate and measure the molecular weights of compounds ranging from small molecules and peptides of less than 1,000 Da up to proteins of 500 kDa. Quantifiable differences in protein/peptide patterns can be statistically evaluated using automated computer programs that represent each protein/peptide measured in the biofluid as a coordinate in multi-dimensional space. This approach has been most successful in the field of clinical biomarker discovery (for example, see [5–7]) as it can be used as a diagnostic tool without needing to know the biomarker's identity. The SELDI system also has the capability to run hundreds of samples in a single experiment. In addition, all the signals from SELDI mass spectrometry are derived from native proteins/peptides (unlike some other proteomics technologies requiring protease digestion), thus directly reflecting the underling physiology of a given condition.

In this study, we undertook an extensive protein/peptide–profiling analysis of cerebrospinal fluid (CSF), investigating a total of 179 CSF samples (90 controls, 58 first-onset, drug-naïve schizophrenia patients, 16 depression patients, five patients with obsessive-compulsive disorder [OCD], and ten patients with Alzheimer disease) using SELDI mass spectrometry in combination with computerized pattern-recognition analysis. We found highly significant and reproducible differences in samples obtained from first-onset, drug-naïve patients with a diagnosis of paranoid schizophrenia as compared to age-matched controls. However, as no samples from patients with non-schizophrenia psychosis were included in the analysis (with the exception of three samples from patients with psychotic depression), no clear conclusions can be drawn with regards to the schizophrenia-specificity of the biomarkers as compared to a first-onset psychosis signature.

## Materials and Methods

### Clinical Samples

The Ethical Committee of the Medical Faculty of the University of Cologne reviewed and approved the protocol of this study and the procedures for sample collection and analysis. All study participants gave their written informed consent. All clinical investigations were conducted according to the principles expressed in the Declaration of Helsinki. CSF and serum samples were collected from drug-naïve patients diagnosed with first-episode paranoid schizophrenia or brief psychotic disorder due to duration of illness (DSM-IV 295.30 [*n* = 54] or 298.8 [*n* = 4], total *n* = 58), from 16 patients diagnosed with major depressive disorder (DSM-IV 296.22–4; 296.33–4), from five patients with OCD (DSM-IV 300.3), and from demographically matched healthy volunteers (*n* = 80) (Tables 1 and 2). Fresh-frozen prefrontal cortex tissue (Brodmann area 9) from gray matter of eight schizophrenia and eight well-matched control individuals was obtained from the Neuropathology Consortium of the Stanley Brain Collection (Stanley Medical Research Institute, http://www.stanleyresearch.org). A detailed history of antipsychotic drug use of included patients is listed in Tables S1 and S2.

**Table 1:**
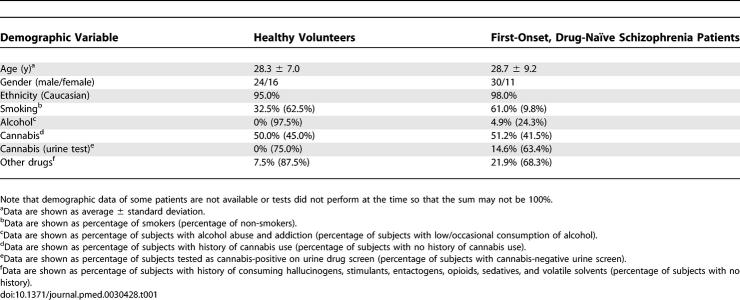
Demographic Details of Subjects in the First CSF SELDI Experiment

**Table 2:**
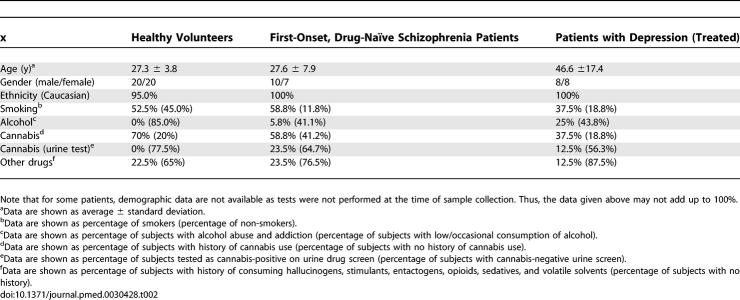
Demographic Details of Subjects in the CSF Validation Sample Set

### Preparation of CSF Samples for SELDI Analysis

An amount (5 μl) of each CSF sample was applied to protein chips with different chemical properties at various pH conditions. The best condition was chosen at pH 9.0 on strong anion exchanger Q10 chips, based on number and separation of peaks resolved. Briefly, the array spots were pre-activated twice with binding buffer (100 mM Tris-HCl [pH 9.0]) at room temperature for 10 min on a shaker (frequency = 600 rpm). An amount (5 μl) of binding buffer was added to each spot prior to the addition of 5 μl of CSF sample. The protein chips were incubated on a shaker for 60 min at room temperature, then washed twice with binding buffer and once with H_2_O, and air-dried. The chips were then sequentially treated twice with 0.6 μl of a 100% saturated sinapinic acid (3,5-dimethoxy-4-hydroxycinnamic acid) in 50% acetonitrile and 0.5% trifluoroacetic acid. The chips were analyzed with the Ciphergen ProteinChip Reader (Ciphergen ProteinChip System Series 4000, Ciphergen Biosystems, http://www.ciphergen.com). Each sample was analyzed twice to confirm reproducibility in identifying the differentially expressed proteins and to ensure stability of instrument performance.

### SELDI–Time-of-Flight Mass Spectrometer Analysis

The arrays were analyzed with the Ciphergen ProteinChip System Series 4000 (Ciphergen Biosystems). Mass spectra of proteins were generated by using an average of 254 laser shots at a laser intensity of 1,800 arbitrary units. For data acquisition, the detection size ranged between 2.5 and 200 kDa. The laser was focused at 10 kDa. The mass-to-charge ratio *(m/z)* of each of the proteins captured on the array surface was determined relative to external calibration standards (Ciphergen Biosystems): bovine insulin (5,733.6 Da), human ubiquitin (8,564.8 Da), bovine cytochrome c (12,230.9 Da), bovine superoxide dismutase (15,591.4 Da), horseradish peroxidase (43,240 Da), and BSA (66,410 Da). The data were analyzed with ProteinChip data analysis software version 3.0 and Ciphergen Express Software 3.0 (Ciphergen Biosystems). Matrix attenuation was set at 2,500 Da, and shot sequence was set with one warming shot at 2,000 nJ followed by four shots at 1,800 nJ. The Ciphergen Express Software 3.0 was used to compile all spectra and to autodetect quantified mass peaks within a mass range of between 2,500 and 200,000 Da, and the signal-to-noise threshold was set at 5. Peak labeling was completed using second-pass peak selection with 0.2% of the mass window, and estimated peaks were added. The peak information of all spectra was exported for further statistical analysis.

### Peptide and Protein Identification

Typically, 10-μl CSF samples from both the control and schizophrenia groups were applied to Q10 protein chips at pH 9.0 (50 mM Tris-HCl). Proteins/peptides bound to the chip were eluted with 5 μl of elution buffer (30% acetonitrile, 50 mM sodium acetate [pH 3.0]) and desalted using a C18 Ziptip (Millipore, Billerica, Massachusetts, United States) according to the manufacturer's instructions. The peptides were eluted with 0.1% formic acid/50% aqueous acetonitrile (2 μl) and were further examined by MALDI mass spectrometry for the confirmation of the enriched 3,959-Da peak in the CSF sample of the patients with schizophrenia. A CSF sample with a high 3.959 peptide level was also loaded onto a C18 nano-column for online LC-ESI-MS/MS on a QSTAR quadrupole-TOF mass spectrometer (Applied Biosystems, Foster City, California, United States) for de novo sequencing.

For identification of protein biomarkers, CSF proteins were purified from pooled CSF by a combination of anion exchange chromatography (HyperD, Ciphergen Biosystems) followed by SDS-PAGE. CSF protein (~1 mg) was diluted in two volumes of buffer (50 mM Tris-HCl [pH 9]) and applied to the HyperD column equilibrated with the same buffer. The bound protein was then eluted by step gradients using pH 7.0, 6.0, 5.0, 4.0, and 3.0 buffers (Expression Difference Mapping Kit, Ciphergen Biosystems). The fractions were concentrated using Centricon YM10 (Millipore) to ~20 μl, followed by SDS-PAGE analysis (NuPAGE Bis-Tris gel, 4%–12%, Invitrogen, Carlsbad, California, United States). The band expected to correspond to the SELDI peak was cut from the gel. Approximately one third of the excised gel band was used for passive elution of the protein followed by SELDI analysis to confirm that the correct protein had been isolated. The remaining two thirds of the gel band was in-gel digested with trypsin (1:50; Promega, Madison, Wisconsin, United States) overnight at room temperature. The resulting peptide mixtures were then analyzed by LC-ESI-MS/MS (QSTAR, Applied Biosystems), and the protein was identified by database searching using Mascot software (Matrix Science, London, United Kingdom).

*S*-cysteinylated or *S*-glutathionylated isoforms of proteins were confirmed by comparing the spectra before and after on-chip reduction using β-mecaptoethanol. In brief, CSF protein and peptide binding was performed as described above and, in the final step, each spot was washed with 100 μl of 1 mM HEPES [pH 7.5]. The proteins and peptides on the chips were then reduced with 1/40 β-mecaptoethanol (1 μl) for 30 min at room temperature. Water (1 ml) was added onto each spot and evaporated. This procedure was repeated twice. Matrix was then added, and data were acquired using ProteinChip Reader (Ciphergen ProteinChip System Series 4000).

Quantitative analysis of transthyretin in human serum samples was carried out by enzyme-linked immunosorbent assay (ELISA). Samples were defrosted from −80 °C and mixed on a benchtop vortexer for 10 min before experimental work commenced. All samples were assayed blind to the clinical conditions. The identities of all subjects were kept blind by code numbers until all biochemical analyses had been completed.

Controls and patient-derived human serum samples were diluted 1,000 times with PBS (pH 7.4) (Sigma, St. Louis, Missouri, United States), and 100 μl of each diluted sample was loaded onto ELISA Maxisorb plates (Nunc, http://www.nuncbrand.com) together with transthyretin standards (Sigma) before being incubated for 1 h. All samples were tested in duplicate. After washing with washing buffer (0.03% Tween 20 in PBS), the plates were blocked with 100 μl of 5% skimmed-milk powder for 60 min. Transthyretin antibody (100 μl) (DakoCytomation, Glostrup, Denmark, 1:500 diluted in 2.5% skimmed-milk powder) was incubated in 96-well plates for 60 min. The plates were washed four times with washing buffer followed by the addition of 100 μl of secondary antibody (anti-rabbit HPP-linked IgG [Cell Signaling Technology, Beverly, Massachusetts, United States] 1:2000) to each well and incubated for 60 min. After washing with washing buffer three times, 100 μl of substrate (TMB One solution, Promega) was added into each well, and the mixture was incubated at room temperature for 10 min. The plate was read at 450 nm (Model 680, Bio-Rad, Hercules, California, United States).

### Western Blot Analysis

The preparation of human brain samples for Western blot analysis and the details of performing Western blotting were as described previously [8]. In brief, equivalent amounts of protein (30 μg per sample) were resolved by electrophoresis on 10% polyacrylamide gels and transferred onto nitrocellulose, which was then incubated with primary antibody (anti-transthyretin [DakoCytomation] 1:1,000) or anti-VGF (Santa Cruz Biotechnology [Santa Cruz, California, United States] 1:500) in 3% milk–PBS overnight at 4 °C, followed by incubation of a secondary antibody (HRP-conjugated anti-rabbit secondary antibody [Cell Signaling Technology, 1:2500]) at room temperature for 1 h. Enhanced chemiluminescence (LumiGlu, Cell Signaling Technology) was used to detect signals from the blot. Consistency of protein loading and transfer was determined by Ponseau S staining.

### Statistical Analysis

Multivariate statistical analysis techniques including principal component analysis (PCA), partial least-squares discriminate analysis (PLS-DA), and partial least-squares (PLS) were employed to summarize the data output from Ciphergen Express. Holdout cross-validation was performed three times so that the sensitivity and specificity of the PLS model could be estimated. In each of the three rounds of holdout cross-validation, one third of the samples were randomly selected to form the validation data and the remaining samples were used as the training data. All multivariate analyses were performed using SIMCA-P+ 10 (Umetrics, http://www.umetrics.com). Sensitivity is defined as the proportion of true positives that it detects of all the positives, and specificity is defined as the proportion of true negatives that it detects of all the negatives. Where appropriate, a *t*-test was performed using the Statistical Package for Social Scientists (SPSS/PC+; SPSS, Chicago, Illinois, United States). Correlation analysis was performed using Matlab 7.0.1 (Mathwork, Natick, Massachusetts, United States). Two-way analysis of variance (ANOVA) was performed using the “ANOVA” function from the CAR package in R (R project, http://cran.r-project.org). Type III sums of squares was used as this study had an unbalanced experimental design.

## Results

### Alterations of CSF Protein/Peptide Profiles in First-Onset, Drug-Naïve, Paranoid Schizophrenia Patients

**Figure 1:**
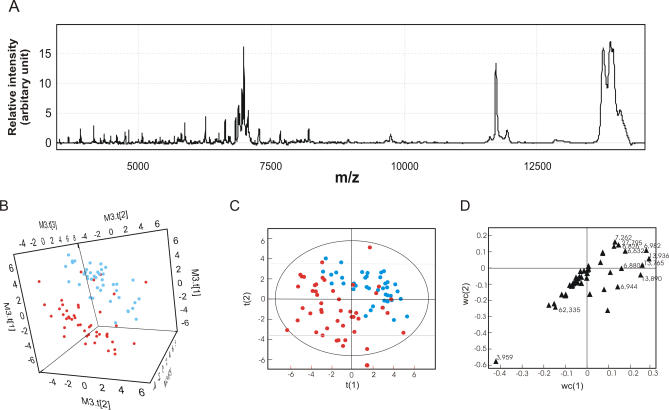
Protein/Peptide Profiling of CSF Samples from First-Onset, Drug-Naïve Schizophrenia Patients Using SELDI Mass Spectrometry (A) Typical CSF protein/peptide spectrum using an anion exchanger chip (Q10; 50 mM Tris-HCl [pH 9.0]) showing the *m/z* range of 2,500–15,000 from a healthy volunteer is shown. (B) The peak intensity of protein/peptide peaks from SELDI spectra were analyzed using PCA and PLS-DA models. A 3D PLS-DA scores plot indicates clusters of healthy volunteers (in blue) and untreated, drug-naïve schizophrenia patients (in red). (C) and (D) PLS-DA scores and loadings plots. The scores plot is similar to (B) but only the first two components were used to discriminate between healthy controls and patients. The loading plot as shown in (D) indicates the key protein/peptide peaks contributing the most towards the separation as shown in (C). The 3,959-Da VGF23–62 peptide was found to predominantly contribute to the separation between the two groups.

In a first set of experiments, we examined protein/peptide profiles of CSF samples from 41 first-onset, drug-naïve, paranoid schizophrenia patients and 40 demographically matched healthy volunteers using SELDI mass spectrometry. CSF proteins and peptides were profiled using Q10 (strong anion exchanger) chips at pH 9.0. An example of the CSF protein/peptide profile of a healthy volunteer is shown in Figure 1A. Approximately 75 peaks can be readily detected with a signal-to-noise ratio of >5 under this binding condition. Plots of PLS-DA scores based on SELDI spectra of CSF samples showed a clear differentiation between healthy volunteers and drug-naïve patients with first-onset, paranoid schizophrenia (Figure 1B and 1C). Similar results were found using PCA (unpublished data). The loading coefficients indicated that the peak with an *m/z* of 3,959 contributed the most to the separation of the two groups (Figure 1D).

**Figure 2:**
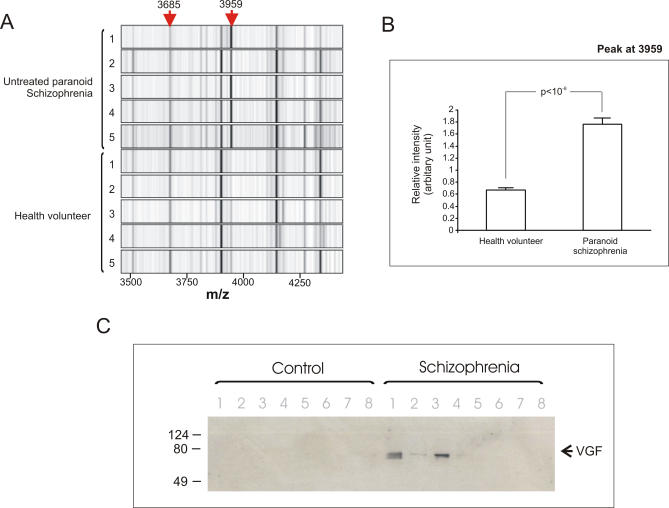
Up-Regulation of *m/z* = 3,959 VGF23–62 Peptide Peak in CSF from First-Onset, Drug-Naïve Schizophrenia Patients Forty healthy volunteers and 41 untreated paranoid schizophrenia CSF samples were included in this study. A clear up-regulation of the peak at 3,959 Da was observed (as shown in [A], with five representative spectra from each group). Figure 2B reveals the relative intensity of a 3,959-Da peak in healthy volunteers and first-onset, drug-naïve schizophrenia patients. Figure 2C shows a Western blot analysis of VGF protein in the prefrontal cortex of age-matched controls and patients with schizophrenia. Eight schizophrenia and eight control brains (prefrontal cortex, age matched) were randomly selected, and Western analysis was performed using a polyclonal antibody against the C-terminal region of the VGF protein. VGF was found up-regulated in four patients with schizophrenia (out of eight patients), while no signal was detected in the control patients. This experiment was repeated twice on the same samples under identical conditions, except that a different batch antibody from the same company was used. Similar results were obtained. For demographic details, see Table 4.

Further analyses showed a 2.8-fold elevation of an *m/z* = 3,959 peptide in first-onset, drug-naïve, paranoid schizophrenia patients as compared to the demographically matched control group (*p* = 10^−8^, *t*-test; Figure 2). In addition, the loading plot also showed significant reductions in peaks of *m/z* = 23,490 and clusters of peaks between *m/z* = 13,600–14,000 and *m/z* = 6,800–7,300 (~20% decreases, *p* < 0.01, *t*-test), all of which also contributed to the separation between classes. We have identified the proteins and peptides at *m/z* = 3,959 and in the cluster of *m/z* = 13,600–14,000. The *m/z* = 6,800–7,300 biomarkers are likely to be influenced by the doubly charged ion signals of the *m/z* = 13,600–14,000 protein cluster and may only reflect the altered levels in *m/z* = 13,600–14,000 (Figure S1). The sensitivity and specificity of this model based on holdout cross validation was 80% and 95%, respectively (Table 3; see Materials and Methods). These alterations were not affected by most demographic variables including age, gender, alcohol, and illicit drug use, although we identified a correlation with smoking (Table S3). However, a disease-associated increase in VGF levels remains significant after controlling for smoking effects using a two-way ANOVA (Table S3.5).

**Table 3:**
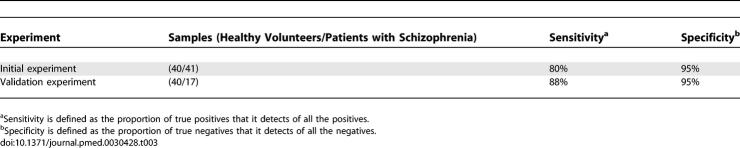
Sensitivity and Specificity of PLS Models Calculated from the Two Independent Experiments

**Table 4:**
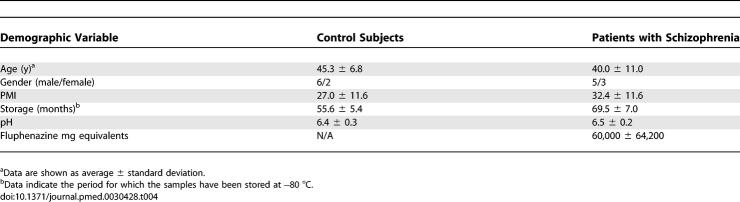
Demographic Details of Subjects Investigated for VGF Expression by Western Blot Analysis of Post-Mortem Brain

### Identification of the 3,959-Da Peptide as a VGF Fragment

**Figure 3:**
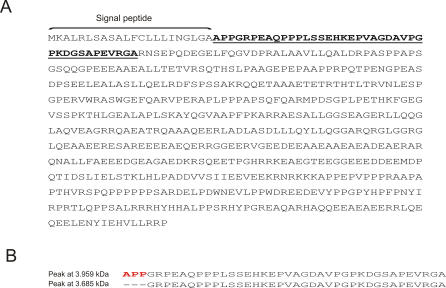
Mapping and Identification of the Biomarker Peptide Derived from the Native VGF Protein The 3,959-Da “schizophrenia” peptide was mapped to amino acids 23–62 of the native VGF protein (in bold; underlined), immediately next to a predicted secretory signal peptide (using InterProScan: http://www.ebi.ac.uk/cgi-bin/iprscan). Another native VGF-derived peptide did not show differential expression between healthy volunteers and patients with schizophrenia (as shown in Figure 2A). This peptide has an identical sequence except that it is three amino acids shorter at the N-terminus compared to the “disease-specific” VGF peptide.

The 3,959-Da peptide was purified using identical conditions as employed in the profiling experiment followed by C18-Ziptip purification (see Figure S2). The eluted proteins and peptides from Ziptips were subjected to MALDI to confirm the mass, and the peak was identified as a 40-amino acid peptide using de novo sequencing (Figure S2). This peptide sequence was mapped to amino acids 23–62 of the native VGF protein (in bold underlined, Figure 3A), a neurosecretory protein known to regulate metabolism [9] and synaptic plasticity [10], and is immediately next to a predicted secretory signal peptide. Interestingly, another VGF-derived peptide with an *m/z* of 3,690 in CSF that did not appear to differ between control and schizophrenia patients was identified (*p* = 0.85, *t*-test). This peptide showed the same peptide sequence as the 40-amino acid VGF peptide except that three amino acids were absent at the first N-terminal (Figure 3B; de novo sequencing data not shown). This indicates that the 40-amino acid VGF peptide with the “APP” sequence in the amino terminus may have specific functions and/or may be linked to the pathophysiology of schizophrenia.

### Increased VGF Protein Expression in Post-Mortem Brain Tissue from Patients with Schizophrenia

To examine whether VGF protein expression is also altered in schizophrenic post-mortem brain tissue, we performed Western blot analysis on gray matter in prefrontal cortex tissue from eight patients with schizophrenia and eight demographically matched control subjects. Western blot analysis using an antibody recognizing the C-terminal sequences of VGF showed a strong expression in four out of eight patients while, in control brains, the level of VGF was below the detection level (Figure 2C).

### Identification of the 13.6–14-kDa Protein Cluster as Transthyretin

**Figure 4:**
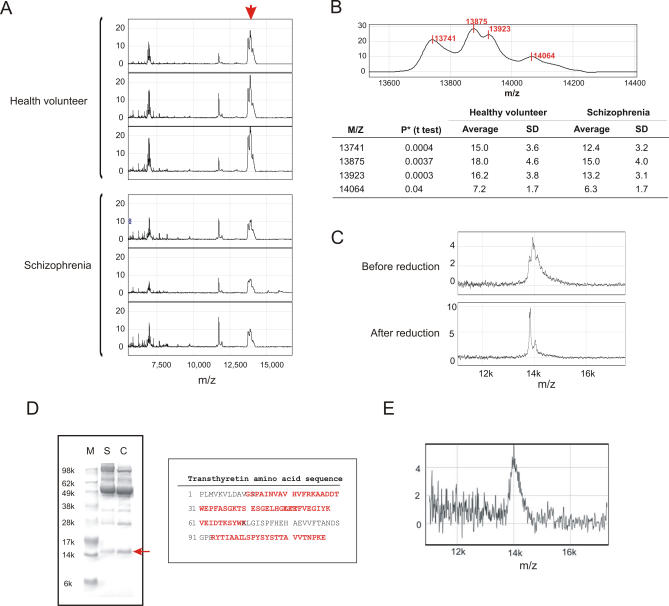
Down-Regulation of Three Different Forms of Transthyretin in CSF from First-Onset, Drug-Naïve Schizophrenia Patients Examples of CSF spectra from healthy volunteers and patients with schizophrenia within 6–17 kDa are shown in (A). The peak cluster indicated (red cursor) is enlarged in (B). Statistical details of each sub-peak are listed in the table presented just below (B). On-chip reduction of CSF peptide/protein with β-mecaptoethanol at room temperature showed that the three peaks 13,741, 13,875, and 13,923 Da were reduced into a single peak (C), suggesting that they are different *S*-cysteinylated derivatives of the same protein. To identify the protein, CSF samples from a healthy volunteer and a patient with schizophrenia were applied to an anion exchanger column (HyperD, Ciphergen Biosystems) and eluted with pH 9–pH 3 buffers. A major ~14–15-kDa band was eluted in the pH 3 fraction (D, left panel). The protein was identified as transthyretin using LC-MS/MS (D, right panel) and the sequence coverage is shown in red. Note, that all the peptides shown have an *E-*score of < 0.05, indicating a high probability for the assigned protein identity. In addition, the band was confirmed to be the peak cluster at around 13.5–14 kDa in the spectra by eluting the protein(s) from the gel and SELDI analysis (E).

The protein cluster between 13.6 and 14.1 kDa contains four peaks (Figure 4A and 4B), three of which were consistently down-regulated in CSF from first-onset, drug-naïve schizophrenia patients (*p* < 0.01; Figure 4B, lower panel). Studies have suggested that these peaks may be from *S*-cysteinylated derivatives of transthyretin protein [11,12], a thyroid hormone–binding protein that transports thyroxine from the bloodstream to the brain. On-chip reduction of CSF peptide/protein performed using β-mercaptoethanol at room temperature showed that the three peaks *m/z* = 13,741, 13,875, and 13,923 were reduced to a single peak (Figure 4C), confirming that they are derived from the same protein. To determine the protein's identity, a pair of CSF samples from a healthy volunteer and a patient with schizophrenia were applied to an anion exchanger column (HyperD, Ciphergen Biosystems) and eluted with pH 9–pH 3 buffers. A major band (~13–15 kDa) was eluted in the pH 3 fraction (Figure 4D, left panel). The band was confirmed to be the peak cluster at around 13.6–14 kDa in the SELDI spectrum by eluting the protein from the band and running on an NP20 chip to match the mass (Figure 4E). This protein was then digested with trypsin and sequenced using LC-MS/MS. The protein was identified as transthyretin (Figure 4D, right panel).

### Down-Regulation of Transthyretin in Serum Samples from the Same Subjects as well as Prefrontal Cortex Tissue from Patients with Schizophrenia

**Figure 5:**
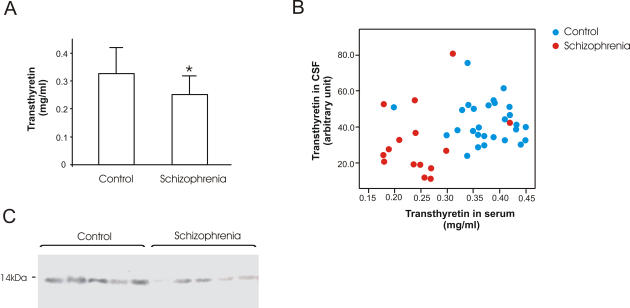
Transthyretin Levels in Sera of First-Onset, Drug-Naïve Schizophrenia Patients and Prefrontal Cortex Post-Mortem Tissue from Schizophrenia Patients The serum samples from the same patients whose CSF protein profiles were measured in Figures 1 and 6 were included in this study. Figure 5A shows serum tranythyretin levels in patients with schizophrenia significantly decreased by ~15% compared to control subjects. Data are shown in mean ± standard deviation, and the asterisk denotes *p* = 0.0007 (*t*-test). Figure 5B indicates no correlation between serum transthyretin and CSF SELDI signals from one of the transthyretin isoforms (*m/z* = 13,741) in the second sample set (for demographics, see Table 2). Similar results were found when comparing with signals from other isoforms in CSF (unpublished data). Figure 5C shows an ~40% decrease of transthyretin expression in prefrontal cortex of five patients with schizophrenia and in five control subjects. For demographic details, see Table 5.

It has been estimated that 3% of transthyretin in the ventricular CSF and 10% of the transthyretin in lumbar CSF are derived from blood [13]. To evaluate the contribution of blood transthyretin to the changes found in CSF in patients with schizophrenia, we also investigated serum transthyretin levels taken from the same individuals (at the same time as the CSF was collected) on whom we had reported in this study (for demographics, see Tables 1 and 2) using ELISA. We found a moderate, but significant, decrease in transthyretin levels in sera from patients with schizophrenia compared to controls (15% decrease, *p* = 0.0007, *t*-test) (Figure 5A). However, we found no correlation between CSF and serum transthyretin from the same individuals, suggesting that the transthyretin level is regulated independently in CSF and serum (Figure 5B). Interestingly, we found an ~40% down-regulation of transthyretin levels in post-mortem prefrontal cortex from patients with schizophrenia compared with controls using Western blot (Figure 5C).

**Figure 6:**
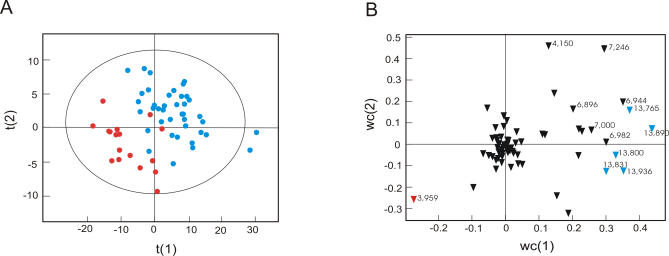
PLS-DA Analysis of CSF Protein/Peptide Profiles from an Independent Validation Sample Set Containing 17 First-Onset, Drug-Naïve Schizophrenia Patients and 40 Healthy Volunteers The demographic details of this sample set are listed in Table 2. The PLS-DA scores plot shows a separation between patients (in red) and healthy volunteers (in blue). The loadings plot indicates similar key variables (e.g., the 3,959-Da VGF23–62 peptide [in red] and transthyretin protein signals between 13,600 and 14,000 [in blue]) identified in the first experiment (see Figure 1).

**Table 5:**
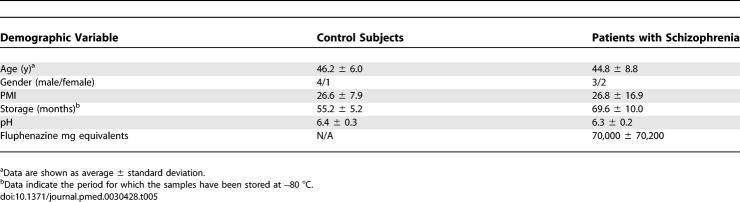
Demographic Details of Subjects Investigated for Transthyretin Expression by Western Blot Analysis of Post-Mortem Brain

### Validation of Protein/Peptide Biomarkers in an Independent Sample Set

We validated our biomarker model in Figure 1 using an independent sample set comprising a further 17 first-onset, drug-naïve schizophrenia patients and 40 demographically matched healthy volunteers. These samples were run using identical conditions as in the previously described experiment. PLS-DA scores and loadings plots showed a very similar result as found in Figure 1 with predominant changes in *m/z* = 3,959, the cluster of 6,800–7,300-Da peptides (likely influenced by the doubly charged ions from transthyretin proteins), and the cluster of 13,600–14,000 proteins (Figure 6). This suggests that these identified alterations in CSF proteins and peptides are a consistent finding and thus may reflect genuinely the early pathophysiology of schizophrenia. The sensitivity and specificity of this model were 88% and 95%, respectively (Table 3). As with the results from the first study, we did not find an influence of most demographic variables on these alterations, with the exception of a potential effect of cannabis use (urine-positive drug screen) on CSF transthyretin levels (*p* = 0.08; Table S3). However, as for the smoking effect, disease-associated changes in transthyretin levels remain significant after controlling for cannabis use using a two-way ANOVA (Table S3.6–S3.8).

### Disease Specificity of the Schizophrenia Biomarker Panel

**Figure 7:**
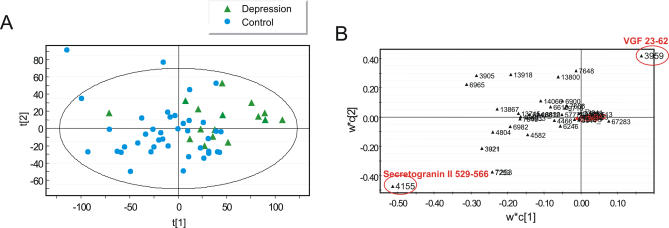
CSF Proteomic Profiles of Depression Patients CSF samples from 16 patients with depression (green triangles), together with 40 healthy volunteers (blue dots), were analyzed with SELDI-MS using Q10 anion exchanger protein chips at pH 9 (50 mM Tris-HCl). (A) The scores plot shows a partial separation between controls and patients with schizophrenia. (B) The loading plot indicates VGF23–62 and a depression-specific secretogranin II 529–566 peptide to be the key protein/peptide peaks contributing the most towards the group separation.

As many psychiatric disorders and neurological diseases share similar symptoms with schizophrenia, it is crucial to test the disease specificity of these protein/peptide biomarkers. We therefore examined CSF samples of three additional psychiatric/neurological disorders, namely depression, OCD, and Alzheimer disease. CSF samples from 16 patients with depression and five patients with OCD, together with CSF samples from 40 healthy volunteers, were analyzed with SELDI-MS using the condition described previously. We found a partial separation between controls and patients with depression (Figure 7A); the key variables contributing to the separation were the above-mentioned VGF23–62 peptide and a secretogranin II peptide 529–566 (Figure 7B; sequencing data not shown). No difference was found between controls and the five patients with OCD (unpublished data). These results indicate that the VGF peptide alone may not be a specific marker for a given psychiatric disease (possibly due to overlapping disease processes, which are not least implied by the fact that patients with a family history of affective disorder have an increased risk for developing schizophrenia, and vice versa). Our data thus suggest that disease specificity may be achieved with a panel of biomarkers (Table S4).

As three out of 16 depression patients presented with psychotic symptoms, we analyzed the psychotic and non-psychotic subgroups separately to examine whether the identified biomarkers are specific for schizophrenia or merely for acute psychosis. No differences could be observed between these two subgroups, and the psychotic subgroup did not co-cluster with schizophrenia patients, suggesting that VGF23–62 and transthyretin peptide/protein changes are biomarkers for schizophrenia rather than acute psychosis (Figure S3). However, as the sample size of patients with psychotic depression was very small, the specificity of the biomarker signature will need to be confirmed on a larger set of samples from patients with psychotic depression, bipolar affective psychosis, and other neuropsychiatric illnesses that present with psychotic states.

In addition, we also analyzed CSF samples from ten patients with Alzheimer disease and from ten age-matched and sex-matched controls using identical experimental conditions. No significant differences between the two groups were identified, although some clear alterations were found using other experimental conditions (unpublished data).

## Discussion

Initial analysis of SELDI spectra of a total of 81 CSF samples (41 from patients with schizophrenia, and 40 from controls) showed a differential distribution of samples from drug-naïve patients with first-onset paranoid schizophrenia away from healthy volunteer samples (Figure 1B–1D). The protein/peptide profile of CSF was found to be characteristically altered in paranoid schizophrenia patients, with the key alterations being the prominent up-regulation of a 40-amino acid VGF peptide and the down-regulation of transthyretin at ~14 kDa along with a peptide cluster at ~6,800–7,300 Da that appeared co-regulated with the transthyretin cluster. These schizophrenia-specific protein/peptide changes were replicated/validated in an independent sample set (*n* = 58) using identical conditions (Figure 6). Both experiments achieved an astonishingly high specificity (rate of true negative) of 95% and a sensitivity of 80% or 88% in the initial study and in the subsequent validation study, respectively (Table 3). This means that virtually no control samples clustered with the schizophrenia group (Figure 1B and 1C). For a high diagnostic validity and consequent therapeutic interventions, an accurate identification of those individuals who truly have the disease is most critical. Correlation analyses suggest that these key alterations are not due to sex, gender, alcohol, smoking, or illicit drug use.

We also investigated the disease specificity of this biomarker panel with a smaller number of CSF samples from patients with depression (including three patients with acute psychotic depression), OCD, and Alzheimer disease. Interestingly, depression and schizophrenia share an increase in the VGF23–62 peptide expression, while transthyretin protein levels were not found to be significantly altered in depression (although we observed a trend towards down-regulation). Indeed, depression samples were most prominently characterized by a distinct decrease of a secretogranin II 529–566 peptide. No difference was found between controls and OCD patients (unpublished data). Additionally, we did not find any significant difference between control and Alzheimer disease CSF samples regarding the schizophrenia biomarker panel (unpublished data).

One of the key findings of this study is the up-regulation of a 40-amino acid VGF23–62 peptide in CSF of first-onset, drug-naïve schizophrenia patients and, to a lesser degree, in patients with depression (Figure 7). Interestingly, a 3.69-kDa VGF26–62 peptide with an identical sequence to the VGF23–62 peptide, except for the absence of the first three amino acids at the amino terminus, did not appear to be differentially expressed in CSF from schizophrenia or depression patients compared to healthy volunteers. However, this VGF26–62 peptide was reported to be decreased in frontotemporal dementia [11]. In addition, a down-regulation of a peptide of 4.82 kDa corresponding to a further VGF peptide (containing amino acids 378–398) was found to be changing in amyotrophic lateral sclerosis [14] and Alzheimer disease [15], highlighting the importance of this protein in neurological/psychiatric disorders. The identified 40-amino acid VGF23–62 peptide may have a distinct function in the schizophrenia/depression disease process, may reflect a disease-associated protease/peptidase dysfunction, and/or may imply the presence of pathological processes in neurons since VGF is selectively expressed in neurons in brain, particularly in the hypothalamus (for review see [16,17]). At this point in time, the biological function of this 40-amino acid VGF23–62 peptide is unknown; however, the full-length protein has been linked to synaptic plasticity [10] as well as penile erection through the activation of paraventricular oxytocinergic neurons [18], and the circadian clock [19]. Future studies are required to explore the possible functions of this and other disease-associated VGF peptides and their respective peptidases.

The increased level of the 40-amino acid VGF peptide may indicate an increase of native (full-length) VGF protein and/or the processing of the protein in the brain. In addition, our data showed that VGF expression was increased in half of the schizophrenia post-mortem brains (Figure 2C). As post-mortem samples were collected from chronic patients who had been chronically treated with antipsychotic drugs, we cannot exclude the possibility of medication effects. However, in view of our CSF results from drug-naïve schizophrenia patients (Figure 1), it is likely that the observed changes in VGF protein/peptide expression are a disease-related phenomenon. Studies have shown that VGF is a secreted polypeptide that is selectively expressed by neurons and by several endocrine and neuroendocrine tissues (for review [16,17]), and is transported in dense core vesicles with the VGF-derived processed peptides being released via a regulated secretory pathway [16,17]. Knockout of the *VGF* gene in mice generated a lean, hypermetabolic, hyperactive phenotype, demonstrating that *VGF* may regulate energy balance [9]. The observation of an increase of the VGF peptide in CSF from patients with schizophrenia, therefore, may point to a hypometabolic state in the schizophrenia brain. Whether or not this is linked to “hypofrontality”, a consistent finding showing decreased metabolic activity in the prefrontal cortex during cognitive activation in patients with schizophrenia [20,21], requires further examination.

Our “metabonomics” data derived from the identical samples used in the current study indicates that there is an increase of CSF glucose level in first-onset, drug-naïve schizophrenia patients which was not observed in sera [22]. This further suggests that the schizophrenia brain may be in a hyperglycemic state, possibly linked to deficiencies in glucose metabolism since we found the majority of glycolysis enzymes down-regulated in prefrontal cortex in a study on post-mortem schizophrenia brain [23]. Interestingly, knockout of the *VGF* gene increases insulin sensitivity in animal models [24], suggesting that an increase of VGF may theoretically decrease insulin sensitivity in the brain. This may explain the increase in glucose levels in CSF from first-onset, drug-naïve schizophrenia patients.

In this study, we also observed a moderate, but consistent, decrease in the transthyretin proteins in CSF from first-onset schizophrenia patients. Transthyretin is a thyroid hormone–binding protein that transports thyroxine from the bloodstream to the brain [25]. This protein is expressed at a high rate in the brain choroid plexus, from which it is released into the CSF [25]. In peripheral tissues, it is expressed primarily in liver [13]. Only an estimated 3% of transthyretin in the ventricular CSF and an estimated 10% of the transthyretin in lumbar CSF are derived from blood [13]. Our ELISA results on the serum samples collected from the identical individuals whose CSF were investigated in this study showed that there is an ~15% decrease of transthyretin levels in serum (*p* = 0.0007, *t*-test; Figure 5A). However, there was no correlation between the level of serum transthyretin and SELDI signals from CSF transthyretin, suggesting that liver-derived tranthyretin may not contribute to the down-regulation in CSF (Figure 5B). Experiments perfusing isolated sheep brains showed that all newly synthesized transthyretin was secreted from the choroid plexus towards the ventricles. The synthesis of this protein is required for the transport of thyroxine [26]. Thus, the reduced level of transthyretin in CSF suggests a lower thyroxine transport in brains of patients with schizophrenia. Indeed, our result showing a down-regulation in transthyretin protein in the post-mortem brain tissue from patients with schizophrenia (Figure 5C) further supports this notion

It is noteworthy that thyroid dysfunction is relatively common in patients with schizophrenia [27,28], and indeed with other psychiatric disorders [29], possibly genetically linked to the disorders. In addition, in patients with severe forms of both hypo- and hyper-thyroidism, psychotic symptoms may occur, and the clinical picture frequently resembles that of schizophrenia [30], which may imply that an increase in central nervous system thyroxine function may be linked. Interestingly, long-term administration of clozapine has been shown to induce transthyretin expression in rat hippocampus and cerebral cortex [31], implying that clozapine enhances central nervous system thyroxine function, supporting the clinical relevance of transthyretin in the early pathophysiology of schizophrenia. Transthyretin has also been found to be differentially expressed in CSF of several other neurological and psychiatric diseases. Sullivan et al. found a 9.6% down-regulation of transthyretin levels (*p* = 0.05, *t*-test) in CSF from patients with [32]. This is in keeping with our observation of a trend towards down-regulation of transthyretin signals (Figure 7A). In frontotemporal dementia, Hansson et al. observed increased expression of two isoforms of transthyretin in CSF using 2D electrophoresis. Taken together, our results stress the importance of transthyretin in the pathophysiology of psychiatric and neurological diseases.

In summary, our results suggest that the application of modern proteomics techniques, particularly mass spectrometric approaches, holds the potential to advance the understanding of the biochemical basis of psychiatric disorders and may in turn allow for the development of diagnostics and better therapeutics. Further studies are required to validate the clinical utility and disease specificity of the identified biomarkers.

### Limitations and Clinical Potential of the Findings

One of the key limitations of the present study is that no (or very few) samples from patients with non-schizophrenia psychosis were investigated. The inclusion of samples from patients with psychosis in the context of bipolar disorder would be particularly desirable. However, it represents a great challenge to collect CSF from patients with acute mania (as the patient must give informed consent and must lie still for safe CSF collection).

A larger number of patient samples, ideally collected in different hospital settings, and the inclusion of samples from patients with “prodromal” schizophrenia (most patients with schizophrenia present with vague mental disturbances for months or years prior to developing overt psychosis) as well as patients with chronic schizophrenia, would increase the confidence in the findings and would have prognostic utility. The responsiveness of the biomarker signature to drug treatment would also be of great interest, as this could be used as a surrogate end point in drug-discovery studies and could help to differentiate drug-responders from non-responders and generally may allow for the development of personalized treatment approaches.

The main limitation of the findings with regards to translation into clinical practice is that, in most countries (with the exception of Germany and some Scandinavian countries), CSF is not routinely collected in patients with first-onset psychosis. A CSF-based diagnostic assay would therefore require a sea-change in clinical practice.

The identification of serum biomarkers would be much more desirable, but is technically challenging. However, our laboratory has identified a number of putative serum biomarkers, which are currently being followed up and validated (unpublished data).

Once sensitive and specific markers are established, diagnostic assays can be developed to validate the identified biomarkers on a larger sample set and, ultimately, in the clinical setting. A likely scenario could be a multi-analyte array configuration based on up to ten indicative biomarkers. Such systems will be configured initially into bench-top instruments and, subsequently, into disposable, easy-to-use diagnostic platforms for the physician's office or the patient's bedside based on hand-held readers. The sensors will be designed with an in-built ability to allow the collected data to be electronically transmitted directly to the physician for interpretation.

## Supporting Information

Figure S1 The *m/z* = 6,800–7,000 Region of CSF Spectra Was under a Strong Influence of Doubly Charged Ions from the Transthyretin Proteins (1.8 MB TIF)

Figure S2 Identification of the 3,959-Da Schizophrenia-Specific Peptide as a VGF Fragment (2.6 MB PPT)

Figure S3 Patients with Depression who Presented with Acute Psychotic Symptoms (*n* = 3) at the Time of Sample Collection Did Not Co-Cluster with Schizophrenia Patients and Did Not Show a Significant Difference when Compared to Non-Psychotic Patients with Depression (1.8 MB TIF)

Table S1 History of Antipsychotic Drug Use for Patients Included in Figure 2C (28 KB DOC)

Table S2 History of Antipsychotic Drug Use for Patients Included in Figure 5C (26 KB DOC)

Table S3 Correlation Analyses of CSF Expression Levels of VGF23–62 (*m/z* = 3,959), TTR Proteins (*m/z* = 13,765, 13,853, and 13,936), and Demographic Variables (27 KB DOC)

Table S4 Summary of Potential Peptide/Protein Biomarkers for Four Neurological/Neuropsychiatric Disorders Investigated in This Study (94 KB DOC)

## Abbreviations

ANOVA: analysis of variance
CSF: cerebrospinal fluid
ELISA: enzyme-linked immunosorbent assay
*m/z,*: mass-to-charge ratio
OCD: obsessive-compulsive disorder
PCA: principal component analysis
PLS: partial least-squares
PLS-DA: partial least-squares discriminate analysis
SELDI: surface-enhanced laser desorption ionization
