# Supplementary material for: Disease Biomarkers in Cerebrospinal Fluid of Patients with First-Onset Psychosis
Source: PLoS Med. 2006 Nov 7;3(11):e428. doi: 10.1371/journal.pmed.0030428 (PMC1630717; doi:10.1371/journal.pmed.0030428)
Supplement: Supplementary file 1 [file pmed.0030428.st001.doc]

**Supplementary Table S1. History of antipsychotic drug use for patients included in Figure 2C**

|  | **History of drug use** |
| --- | --- |
| Patient 1 | thioridazine, thiothixene |
| Patient 2 | haloperidol, thiothixene, perphenazine |
| Patient 3 | thioridazine, olanzapine, risperidone |
| Patient 4 | fluphenazine, thioridazine, haloperidol |
| Patient 5 | thiothixene, clozapine |
| Patient 6 | Off approx 1 month: risperidone, fluphenazine |
| Patient 7 | clozapine, haloperidol, fluphenazine, thioridazine, perphenazine, risperidone |
| Patient 8 | fluphenazine, haloperidol, clozapine, risperidone |
