# Supplementary material for: Disease Biomarkers in Cerebrospinal Fluid of Patients with First-Onset Psychosis
Source: PLoS Med. 2006 Nov 7;3(11):e428. doi: 10.1371/journal.pmed.0030428 (PMC1630717; doi:10.1371/journal.pmed.0030428)
Supplement: Supplementary file 3 [file pmed.0030428.st003.doc]

**Supplementary Table S3. Correlation analyses of CSF expression levels of VGF23-62 (m/z=3,959) and TTR proteins (m/z=13,765, 13,853 and 13,936) and demographic variables.**

Effects of demographic variables were examined using Spearman’s rank correlation test. Correlation coefficient and p value (Holm (FWER) corrected) for the training and test sample sets are shown in Table S3.1-3.4, respectively. Demographic variables that were significantly different between the disease and control groups and showed correlation with levels of VGF and TTR proteins were considered as potential confounds. Smoking showed some correlation with VGF23-62 peptide levels in the first SELDI experiment (Table S3.2) whilst there was a trend between the levels of TTR and urine cannabis use (positive on urine drug screen) in the second SELDI experiments (Table S3.4). Two-way ANOVA (type III sum of squares (due to an unbalanced experimental design)) were then performed using “Anova” function from CAR package in R to investigate the effects of smoking and cannabis use on the levels of VGF23-62 and TTR proteins. The effect of disease state on VGF peptide levels remains significant after controlling for the effect of smoking (Table S3.5). Similarly, the effect of disease on TTR protein levels remains significant after controlling for cannabis use (Table S3.6-3.8).

Supplementary Table S3.1. Coefficients of correlations between biomarkers and demographics factors in the training samples set

R=

| **m/z** | **Age*** | **Gender**** | **Smoking**** | **Alcohol**** | **Cannabis**** | **Other** drugs** | **Urine cannabis**** |
| --- | --- | --- | --- | --- | --- | --- | --- |
| **3,959** | 0.05 | -0.17 | 0.35 | 0.27 | 0.15 | 0.19 | 0.15 |
| **13,853** | -0.06 | 0.12 | -0.09 | -0.16 | -0.01 | -0.11 | 0.19 |
| **13,890** | 0.00 | 0.13 | -0.08 | -0.11 | 0.07 | -0.10 | 0.11 |
| **13,936** | -0.07 | 0.14 | -0.08 | -0.04 | 0.07 | -0.14 | 0.04 |

Supplementary Table S3.2. p values of correlations between biomarkers and demographics factors in the training samples set.

P=

| **m/z** | **Age*** | **Gender**** | **Smoking**** | **Alcohol**** | **Cannabis**** | **Other** drugs** | **Urine cannabis**** |
| --- | --- | --- | --- | --- | --- | --- | --- |
| **3,959** | 1 | 0.48 | 0.016 | 0.24 | 0.76 | 0.4 | 0.8 |
| **13,853** | 1 | 0.66 | 0.81 | 0.81 | 1 | 0.66 | 1 |
| **13,890** | 1 | 0.66 | 0.88 | 0.88 | 1 | 0.66 | 1 |
| **13,936** | 1 | 0.66 | 0.88 | 0.88 | 1 | 0.66 | 1 |

Supplementary Table S3.3. Coefficients of correlations between biomarkers and demographics factors in test samples set.

R=

| **m/z** | **Age*** | **Gender**** | **Smoking**** | **Alcohol**** | **Cannabis**** | **Other** drugs** | **Urine cannabis**** |
| --- | --- | --- | --- | --- | --- | --- | --- |
| **3,959** | 0.15 | -0.13 | 0.22 | 0.24 | 0.13 | 0.08 | 0.15 |
| **13,853** | -0.03 | 0.06 | -0.2 | -0.23 | -0.01 | 0.12 | -0.37 |
| **13,890** | 0.13 | -0.02 | -0.10 | -0.24 | 0.01 | 0.22 | -0.38 |
| **13,936** | 0.17 | 0.02 | -0.03 | -0.19 | -0.04 | 0.21 | -0.38 |

Supplementary table S3.4. p values of correlations between biomarkers and demographics factors in test samples set.

P=

| **m/z** | **Age*** | **Gender**** | **Smoking**** | **Alcohol**** | **Cannabis**** | **Other** drugs** | **Urine cannabis**** |
| --- | --- | --- | --- | --- | --- | --- | --- |
| **3,959** | 0.84 | 1 | 0.48 | 0.44 | 1 | 0.76 | 0.35 |
| **13,853** | 0.83 | 1 | 0.51 | 0.44 | 1 | 0.76 | 0.08 |
| **13,890** | 0.84 | 1 | 0.98 | 0.44 | 1 | 0.48 | 0.08 |
| **13,936** | 0.84 | 1 | 0.98 | 0.44 | 1 | 0.48 | 0.08 |

Supplementary table S3.5. Analysis of confounding effect of smoking to VGF23-62 levels in CSF.

|  | Sum of square | Df | F value | Prob>F |
| --- | --- | --- | --- | --- |
| Disease | 2.109 | 1 | 27.84 | 1.73e-06 |
| smoking | 0.1527 | 1 | 2.02 | 0.16 |
| Residual | 4.77 | 63 |  |  |

Supplementary table S3.6. Analysis of confounding effect of urine cannabis results to one of TTR peak at 13.831kDa in CSF.

|  | Sum of square | Df | F value | Prob>F |
| --- | --- | --- | --- | --- |
| Disease | 0.095 | 1 | 6.20 | 0.017 |
| Urine cannabis result | 0.025 | 1 | 1.24 | 0.207 |
| Residual | 0.553 | 36 |  |  |

Supplementary table S3.7. Analysis of confounding effect of urine cannabis results to one of TTR peak at 13.870kDa in CSF.

|  | Sum of square | Df | F value | Prob>F |
| --- | --- | --- | --- | --- |
| Disease | 0.187 | 1 | 14.28 | 0.0006 |
| Urine cannabis result | 0.011 | 1 | 0.87 | 0.36 |
| Residual | 0.472 | 36 |  |  |

Supplementary table S3.8. Analysis of confounding effect of urine cannabis results to one of TTR peak at 13.921kDa in CSF.

|  | Sum of square | Df | F value | Prob>F |
| --- | --- | --- | --- | --- |
| Disease | 0.059 | 1 | 4.03 | 0.05 |
| Urine cannabis result | 0.019 | 1 | 1.31 | 0.26 |
| Residual | 0.528 | 36 |  |  |
