# Supplementary material for: Disease Biomarkers in Cerebrospinal Fluid of Patients with First-Onset Psychosis
Source: PLoS Med. 2006 Nov 7;3(11):e428. doi: 10.1371/journal.pmed.0030428 (PMC1630717; doi:10.1371/journal.pmed.0030428)
Supplement: Supplementary file 4 [file pmed.0030428.st004.doc]

**Supplementary Table S4.** Summary of potential peptide/protein biomarkers for four neurological/neuropsychiatric disorders investigated in this study.

| **Disease** | **Putative CSF biomarker** |
| --- | --- |
| Schizophrenia | VGF23-62, transthyretin proteins |
| Depression | VGF23-62, secretogranin II 529-566 |
| OCD | None identified |
| Alzheimer disease | 3.23kDa, 6.35kDa and 8.59kDa peptides |
